# Variant Class–Specific Phenotypes and Outcomes in Pediatric TBX4‐Associated Pulmonary Hypertension

**DOI:** 10.1002/ppul.71857

**Published:** 2026-10-06

**Authors:** Cara Morgan, Alistair Calder, Matina Prapa, Thivya Sekar, Andrew Constantine, Tomas Woodgate, Annabelle Barnes, Eimear McGovern, Paul Aurora, Rossa Brugha, Sadia Quyam, Andrew Bush, Shahin Moledina

**Affiliations:** ^1^ UK National Paediatric Pulmonary Hypertension Service Great Ormond Street Hospital for Children London UK; ^2^ Department of Radiology Great Ormond Street Hospital for Children London UK; ^3^ St George's University Hospitals NHS Foundation Trust SW Thames Centre for Genomic Medicine London UK; ^4^ Department of Histopathology Great Ormond Street Hospital for Children London UK; ^5^ Department of Cardiology Adult Congenital Heart Disease Unit, Queen Elizabeth Hospital Birmingham Birmingham UK; ^6^ Institute of Cardiovascular Sciences, University of Birmingham Birmingham UK; ^7^ Institute of Cardiovascular Sciences, University College London London UK; ^8^ Department of Paediatric Cardiology Children's Health Ireland at Crumlin Dublin Ireland; ^9^ Department of Paediatric Respiratory Medicine Great Ormond Street Hospital for Children London UK; ^10^ National Heart and Lung Institute and Imperial Centre for Paediatrics and Child Health Imperial College London UK; ^11^ Department of Paediatric Respiratory Medicine Royal Brompton Hospital London UK

**Keywords:** case series, children, interstitial lung disease, lung developmental disease, pulmonary arterial hypertension, small‐patellar syndrome

## Abstract

**Aim:**

To describe the variant class associations with phenotype, natural history, treatment response, and outcomes in children with *TBX4*‐associated pulmonary hypertension (*TBX4*‐PH).

**Methods:**

Retrospective cohort analysis of children < 18 years referred to the National Paediatric Pulmonary Hypertension Service between 2001 and 2025.

**Results:**

Twenty‐one children were identified carrying either a copy number variant (CNV group = 10) encompassing *TBX4* or a single nucleotide variant (SNV group = 11). Distinct phenotypic differences were observed between variant classes. Children in the CNV group were referred at a younger age (*p* = 0.03), more frequently had prolonged oxygen dependence from birth (*p* < 0.01), more severe lung disease (*p* < 0.01) and cyanosis (*p* = 0.04), congenital heart disease (*p* = 0.03), neurodevelopmental disorders (*p* < 0.01), and deafness (*p* = 0.01). Children in the SNV group were less likely to have any lung disease (*p* = 0.03). Histopathology from five children (CNV = 3, SNV = 2) demonstrated predominantly lung growth abnormalities with variable pulmonary vascular remodeling. Twenty of 21 (95%) children received long‐term pulmonary vasodilator therapy, and 9 (43%) received concurrent calcium‐channel blockers. Treatment‐naïve children (*n* = 12) demonstrated stable/improved functional class with therapy initiation. Median follow‐up was 7.1 years (range 0.7–17.2). Transplant‐ or Potts shunt‐free survival at 1 and 5 years was 100% and 94.4% respectively and was worse in the CNV group (Log‐rank *p* < 0.05).

**Conclusions:**

In this large pediatric *TBX4*‐PH cohort, children carrying CNVs had greater multisystem involvement and poorer outcomes than those carrying SNVs, highlighting the importance of the underlying genetic finding for risk stratification and tailored management strategies.

## 1 Introduction

Pulmonary hypertension (PH) in children is a heterogeneous condition associated with significant morbidity and mortality, encompassing pulmonary vascular disease occurring in isolation or in association with congenital heart disease, lung disease, developmental disorders and genetic syndromes [1, 2, 3]. Compared with adults, developmental abnormalities of the pulmonary vasculature and lung have a particularly important role in pediatric PH [4]. Advances in genetic testing have increasingly identified monogenic causes that provide insight into these overlapping pulmonary vascular and developmental phenotypes [5, 6]. Rare pathogenic variants in, or deletions of, T‐box transcription factor 4 (*TBX4*) are now recognized as one of the leading genetic causes of pulmonary hypertension (PH) in children [7, 8, 9]. *TBX4* encodes a transcription factor involved in embryogenesis, notably in hind‐limb formation and lung morphogenesis [10, 11]. Microdeletions on chromosome 17q23, encompassing the *TBX4* gene locus, were first reported in 7 children, 5 of whom had small patellar syndrome, also known as ischio‐coxo‐podo‐patella syndrome (ICPPS), and 3 of whom also had PH [12]. While this early report involved copy number variants (CNVs) subsequent studies have also identified single nucleotide variants (SNVs) in *TBX4* in association with ICPPS, developmental lung disorders and childhood‐onset PH [13, 14, 15, 16, 17], contributing to recognition of a boarder clinical phenotype commonly termed *TBX4* syndrome. Other reported features include deafness and congenital cardiac and neurodevelopmental anomalies [12, 13, 14, 17]; however, in individuals with CNVs encompassing *TBX4*, additional genes within the deleted interval may contribute to these syndromic features.

Herein, we describe the largest single‐centre experience of children with *TBX4*‐assosicated PH (*TBX4*‐PH), characterizing it's phenotype, natural history, treatment response and outcomes and compare differences between children carrying pathogenic CNVs with those carrying rare deleterious SNVs.

## 2 Methods

### 2.1 Study Design and Population

We conducted a retrospective analysis of all children aged < 18 years with *TBX4‐*PH referred to the United Kingdom National Paediatric Pulmonary Hypertension Service (NPPHS) from 1st January 2001 to 1st January 2025. Children were included if they had either a pathogenic or likely pathogenic SNV, defined for the purpose of this study as a variant confined to *TBX4*, including single nucleotide variants, small insertions, deletions or duplications (SNV group), or a pathogenic CNV encompassing *TBX4* at chromosome 17q23 (CNV group). All CNVs identified in this cohort were multigene deletions encompassing *TBX4*. Variant pathogenicity was assigned according to the American College of Medical Genetics and Genomics and Association for Molecular Pathology guidelines [18], with incorporation of updated recommendations from the ClinGen Sequence Variant Interpretation Working Group [19, 20]. The requirement for individual informed consent was waived by the NHS Health Research Authority and associated Research Ethics Committee (REC reference17/LO/0008), under the approval titled “Use of routine Great Ormond Street Hospital data for research,” because the study used anonymized routinely collected clinical data.

### 2.2 Clinical Assessments and Definitions

All children underwent standardized initial assessment, including echocardiography, 6‐minute walk test (when age‐appropriate), and cardiac catheterization with acute vasodilator testing (AVT) using inhaled nitric oxide, where clinically indicated [21, 22]. Acute vasoresponder status was defined by standard criteria [23]. A diagnosis of PH was confirmed by cardiac catheterization, or non‐invasively by multimodal imaging and clinical assessment in children where invasive assessment was not possible. Height, weight, and birthweight were expressed as *Z*‐scores [24, 25]. Following initial assessment, children were reviewed routinely with clinical assessment and echocardiography at approximately 3‐monthly intervals. Response to pulmonary vasodilator therapy was assessed by change in World Health Organisation functional class (WHO‐FC) at the subsequent follow‐up. Lung disease was defined by the presence of respiratory symptoms or signs excluding dyspnea as a common feature of PH (Table S1), hypoxemia, or requirement for respiratory support. Interstitial lung disease (ILD) was diagnosed according to the European Research Collaboration for Children's Interstitial Lung Disease (ChILD‐EU) criteria, integrating clinical features, chest imaging, multidisciplinary review and lung histology where available [26]. Severity of lung disease was assessed using the ChILD‐EU‐adapted respiratory severity score (RSS), as described by Morgan et al. [27]; reproduced in Table S1, where a score of 1 indicates no clinical features of lung disease and a score of 5 indicates the need for pressure‐support ventilation.

### 2.3 Statistical Analysis

Categorical variables are presented as number, *n*, and (%). Continuous variables are presented as mean ± standard deviation. Missing data were not imputed except for forward imputation of previous anthropometric data to allow echocardiographic *Z*‐score calculations. Comparisons between groups were performed using the Student's *t*‐test for normally distributed continuous variables and the Mann–Whitney *U* test for non‐normally distributed or ordinal variables. Categorical variables were compared using Fisher's exact test. A *p* value < 0.05 was considered statistically significant. Kaplan‐Meier survival curves were constructed to determine the composite outcome of all‐cause mortality, lung transplantation, or Potts shunt (referred to as *transplant‐ or Potts shunt‐free survival*). Survival time was taken from diagnosis based on initial assessment at the NPPHS to the time of first composite event, patients were censored at the end of the study period and there was complete ascertainment of outcome. A secondary analysis was restricted to transplant‐free survival to facilitate comparison with previously published UK NPPHS idiopathic PAH cohort data [28]. All statistical analyses were performed using R statistics version 4.5.3 (2026.03.11).

## 3 Results

### 3.1 Genotype and Inheritance

We identified 21 children with 19 distinct heterozygous variants (full details provided Tables S2 and S3). Ten (48%) children carried a CNV, with deletions ranging from 1.4 to 2.2 Mb that encompassed the *TBX4* gene, the remainder carried a SNV. Nine distinct SNVs were identified, including 5 not previously reported. Among children with SNVs, there were 6 frameshift, 2 nonsense, and 1 missense variants. These variants were classified as either pathogenic (*n* = 1) or likely pathogenic (*n* = 8).

At initial assessment, there was a family history of PH in 3 families. During follow up an additional 2 parents and one sibling, all carrying the familial *TBX4* variant, developed PH. Parental testing was performed in 8 children with SNVs, which identified one de novo variant and 7 inherited variants in 6 families. Where data was available, clinical features of ICPPS were observed in all variant‐positive family members. A family history of PH was absent in all children with CNVs and all children whose parents underwent cascade genetic testing were found to have de novo variants (*n* = 6), most likely arising through non‐allelic homologous recombination [12].

### 3.2 Phenotype and Natural History

Baseline phenotypic characteristics are summarized in Tables 1 and S4. Ten (48%) were female, with similar gender balance in both CNV and SNV groups (*p* = 0.49). Children carrying a CNV were diagnosed at a significantly younger age than those carrying a SNV (1.5 ± 1.9 vs. 3.5 ± 2.0 years, *p* = 0.03).

**Table 1:** Baseline clinical, echocardiographic, haemodynamic and pulmonary characteristics and outcomes in children with *TBX4*‐associated pulmonary hypertension.

	All (*n *= 21)	CNV (*n *= 10)	SNV (*n *= 11)	CNV vs. SNV *p*‐value
Demographic and neonatal course				
Female	10 (48)	5 (50)	5 (45)	0.59
Age at 1st assessment, years	2.5 ± 2.2	1.5 ± 1.9	3.5 ± 2.0	**0.03**
NICU admission	14 (67)	8 (80)	6 (55)	0.36
Pneumothorax	9 (42)	5 (50)	4 (36)	0.67
PPHN	12 (57)	7 (70)	5 (45)	0.39
ECMO for PPHN, *n*/*N*	6/12 (50)	4/7 (57)	2/5 (40)	1.00
Supplementary oxygen > 4 weeks	10 (48)	8 (80)	2 (18)	**< 0.01**
Clinical features at referral				
Weight *Z*‐score	−0.90 ± 1.19*	−0.60 ± 1.52	−1.17 ± 0.77**	0.29
WHO FC (*n *= 18)	2.5 [1, 4]	3 [1, 4]	2 [1, 4]	0.85
Echocardiography and haemodynamics				
Right atrial area *Z*‐score (*n *= 15)	0.93 ± 1.51**	−0.08 ± 0.56	1.42 ± 1.60**	0.07
TAPSE *Z*‐score (*n *= 14)	−2.26 ± 2.47*	−2.04 ± 1.03**	−2.34 ± 2.90**	0.84
mPAP, mmHg (*n *= 14)	46 ± 16	49 ± 18	44 ± 16	0.58
PVRi, WU.m^2^ (*n *= 13)	11.2 ± 6.3	11.0 ± 7.4	11.3 ± 6.1	0.94
AVT, *n* positive response/*N* tested	4/11 (36)	3/4 (75)	1/7 (14)	0.09
Pulmonary features				
Lung disease	15 (81)	10 (100)	5 (45)	0.03
ILD	11 (52)	6 (60)	5 (45)	0.67
Respiratory support at 1st assessment	9 (61)	7 (70)	2 (18)	**0.03**
First spirometry (*n *= 12)				
%FEV1	63.4 ± 15.3	53.6 ± 4.1	68.2 ± 16.7	0.12
%FVC	61.4 ± 15.6	51.3 ± 8.1	66.4 ± 16.3	0.12
Extra‐pulmonary features				
ICPPS, *n*/*N* data available	19/19 (100)	9/9 (100)	10/10 (100)	—
CHD	9 (43)	7 (70)	2 (18)	**0.03**
Sensorineural deafness	5 (24)	5 (50)	0	**0.01**
Developmental delay	13 (62)	10 (100)	3 (27)	**< 0.01**
Cognitive impairment, *n*/*N* assessable	6/14 (43)	6/6 (100)	0/8	**< 0.01**
Therapy and outcomes				
Initial therapy strategy: mono/dual/triple	9/7/4	5/3/1	4/4/3	0.85
WHO FC change in treatment naïve				1.0
improved	6/12 (50)	3/6 (50)	3/6 (50)	
stabilized	6/12 (50)	3/6 (50)	3/6 (50)	
Potts shunt/transplant/death	2/1/1	2/1/1	0/0/0	1.0

*Note:* Categorical variables represented as *n* (%), ordinal variables as median [range], and continuous variables as mean ± standard deviation. Statistical comparisons in the final column are between the CNV and SNV groups. For *Z*‐score variables, asterisks indicate comparison with the expected population mean of zero using a one‐sample *t*‐test: *p < 0.05; **p < 0.01.

Abbreviations: %FEV1, % best predicted forced expiratory volume in 1 s; %FVC, % best predicted forced vital capacity; AVT, acute vaso‐responsiveness test; CHD, congenital heart disease; CNV, copy number variant encompassing TBX4 gene; ECMO, extra‐corporeal membrane oxygenation; ICPPS, ischio‐coxo‐podo‐patellar syndrome; ILD, interstitial lung disease; mPAP, mean pulmonary artery pressure; NICU, neonatal intensive care unit; PPHN, persistent pulmonary hypertension of the newborn; PVRi, pulmonary vascular resistance indexed to body surface area; SNV, variant confined to TBX4 including *single nucleotide variant*s, small insertions, deletions or duplications; TAPSE, tricuspid annulus systolic excursion; WHO FC, World Health Organisation functional class.

#### 3.2.1 Neonatal Course

All children were born at term (gestational age > 37 weeks) and none were small for gestational age, with a mean birth‐weight *Z*‐score of −0.16 ± 1.24. Gestational age and birth weight did not differ between the CNV and SNV groups (*p* = 0.81, and *p* = 0.16 respectively). Fourteen (67%) children required admission to a neonatal intensive care unit: 2 (10%) with respiratory distress syndrome and 12 (57%) with persistent pulmonary hypertension of the newborn (PPHN). Nine (43%) neonates developed a pneumothorax. Among children with PPHN, 6 (50%) required extracorporeal membrane oxygenation (ECMO), and 3 (25%) were initially diagnosed with PPHN due to meconium aspiration syndrome. Pulmonary hypertension was considered to have resolved in 6 children with PPHN: 4 by 1 month of age and a further 2 at 7 and 9 months. Ten (48%) children required supplemental oxygen beyond the neonatal period, occurring more frequently in the CNV group than in the SNV group (*p* = 0.01).

#### 3.2.2 Baseline Clinical, Echocardiographic, and Haemodynamic Assessments

At baseline assessment on referral to NPPHS, children demonstrated impaired somatic growth (median weight *Z*‐score −0.90 ± 1.19, *p* < 0.01), reflecting reduced growth from birth (mean difference −0.9 ± 1.2; *p* = 0.01). Most children were symptomatic at presentation (86%), with exertional dyspnea the most common symptom (29%). Cyanosis at rest occurred exclusively in the CNV group (5 of 10 children; 50%), whereas syncope occurred exclusively in the SNV group (4 of 11 children; 36%).

Echocardiographic findings included right atrial dilatation (right atrial area, RAA; mean *Z*‐score 0.93 ± 1.51; *p* < 0.05) and impaired tricuspid annular systolic excursion (TAPSE; mean *Z*‐score −2.26 ± 2.47; *p* < 0.05). No significant echocardiographic differences were observed between the CNV and SNV groups (RAA *Z*‐score, *p* = 0.07 and TAPSE *Z*‐score *p* = 0.84).

Haemodynamic assessment was performed in 14 (67%) children at a median age of 2.9 years (range 0.4 to 6.8), of whom 57% were receiving background therapy. Mean pulmonary artery pressure (mPAP) was 46 ± 16 mmHg and indexed pulmonary vascular resistance (PVRi) was 11.2 ± 6.3 Wood units.m^2^. Acute vasoreactivity testing (AVT) was performed in 11 children, with 4 (36%) meeting criteria for an acute vasodilator response. No significant difference in haemodynamic parameters was observed between the CNV and SNV groups (mPAP *p* = 0.58, PVRi *p* = 0.94, and AVT *p* = 0.09).

#### 3.2.3 Clinical and Radiological Assessment of Lung Disease

At referral 15 (71%) children had clinical features of lung disease. Compared with the SNV group, children in the CNV group were more frequently affected (10 vs. 5; *p* = 0.03), were more likely to require respiratory support (7 vs. 2; *p* = 0.03), and had more severe disease as assessed by the RSS (median 4 vs. 1; *p* < 0.01) (Figure 1). Interstitial lung disease was diagnosed in 11 children (CNV group, *n* = 6; SNV group, *n* = 5; *p* = 0.67). A detailed phenotypic comparison between children with and without ILD is provided in Table S4. Children with ILD were more likely to require supplemental oxygen beyond 4 weeks of age (*p* = 0.02), demonstrated poorer somatic growth (mean weight *Z*‐score −1.23 ± 1.07; *p* < 0.01), and showed reduced growth from birth (mean difference −1.0 ± 1.2; *p* = 0.04) compared with those without ILD. Spirometry was feasible in 5 children with ILD and demonstrated reduced forced vital capacity compared to children without ILD (mean % best predicted 50.3 ± 15.7 vs. 69.3 ± 10.2; *p* = 0.03).

**Figure 1:**
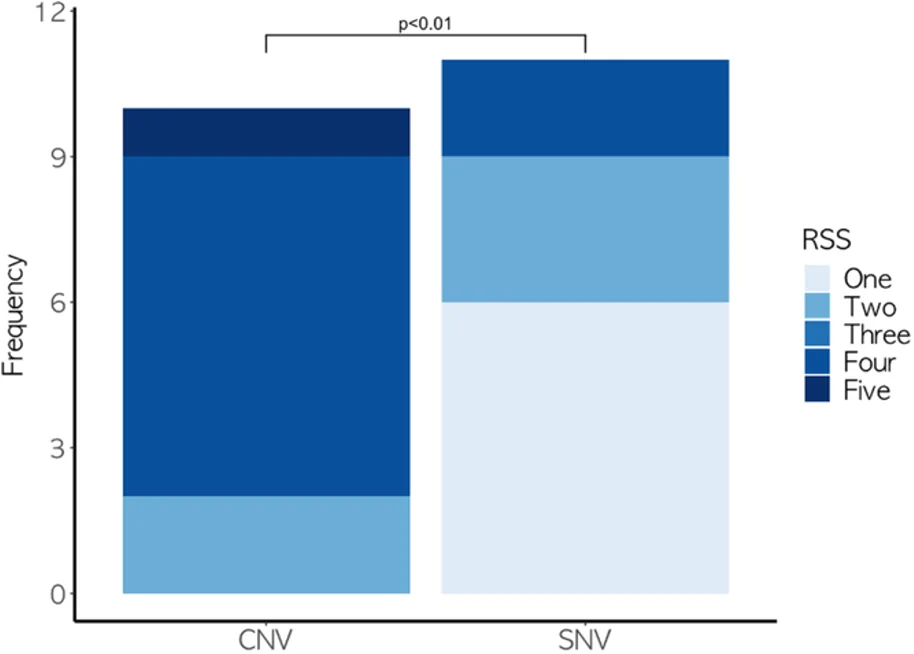
Comparison of baseline respiratory severity scale (RSS) between children with a copy number variant (CNV) and variant confined to *TBX4* including single nucleotide variants, small insertions, deletions or duplications (SNV) using Mann–Whitney *U* test. [Color figure can be viewed at wileyonlinelibrary.com]

All children underwent baseline contrast‐enhanced computed tomography (CT) imaging. Radiological features are summarized in Table S5 and illustrated in Figure 2. The most common abnormality was ground glass opacification (*n* = 13), but with variable distribution (Figure 2a,b), followed by mild bronchial dilatation or wall thickening (*n* = 9) (Figure 2a). Less frequent abnormalities included septal thickening (*n* = 2), vascular tortuosity (*n* = 2), and features suggestive of lung fibrosis (*n* = 1). Overall, radiological features were considered consistent with lung disease in 7 children, equivocal in 6, and not consistent in 8. In children with a diagnosis of ILD, 9 had features on CT consistent with ILD. Diffuse ground glass opacification was observed more frequently in the CNV group than in the SNV group (50% vs. 10%, *p* = 0.06), while no other radiological differences were identified between variant classes.

**Figure 2:**
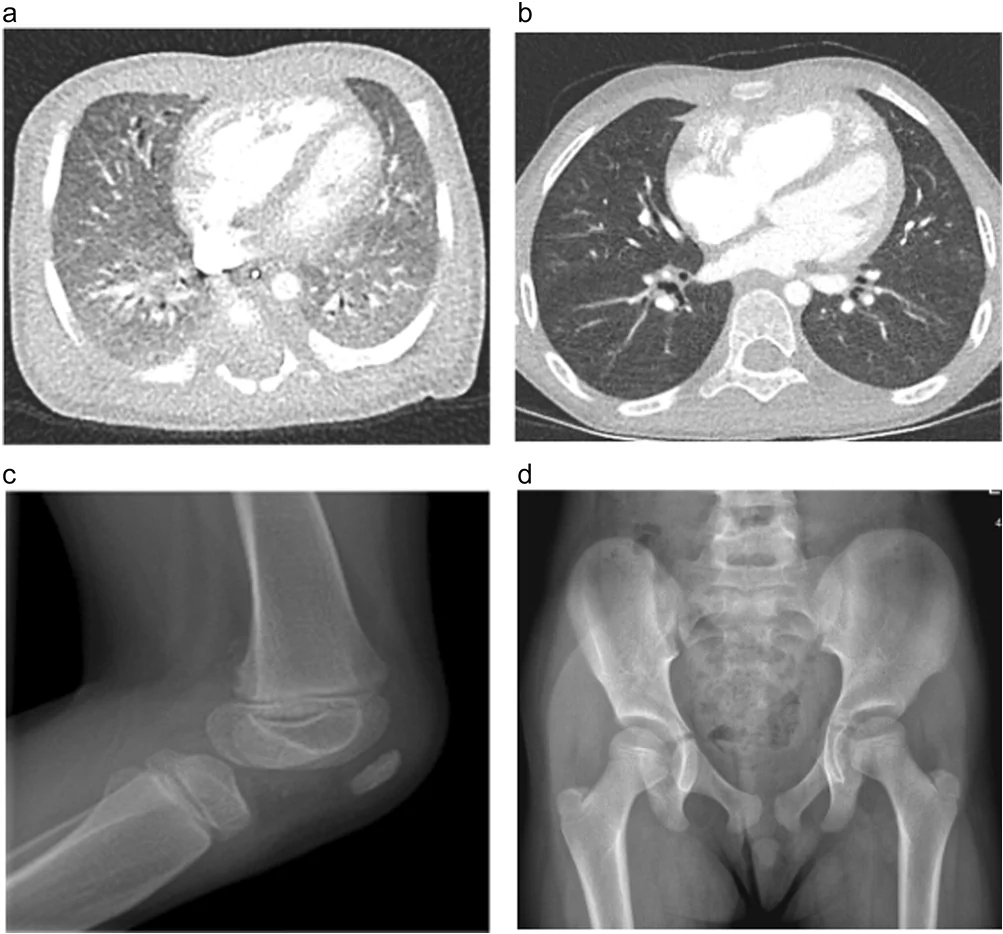
(a) Axial chest computed tomography (CT) scan in a two‐week old child with persistent pulmonary hypertension of the newborn and copy number variant encompassing *TBX4* who was ventilated at the time shows diffuse ground glass opacification and prominent bronchi, with mild bronchial dilatation in the right middle lobe. (b) Axial chest CT in 5‐year‐old with single nucleotide variant (SNV) in *TBX4* and clinical presentation of idiopathic pulmonary arterial hypertension that shows subtle perivascular ground glass opacification, which is a non‐specific finding in pulmonary arterial hypertension. (c) Lateral knee radiograph in 6‐year‐old female with SNV in *TBX4* demonstrates patellar hypoplasia. (d) Anteroposterior radiograph of pelvis in the same patient demonstrates the ischiopubic synchondroses are wide for age and the femoral necks are long.

#### 3.2.4 Extra‐Pulmonary Characteristics

All children in whom data were available had clinical findings and/or radiographic evidence of lower‐limb musculoskeletal abnormalities consistent with ICPPS (*n* = 19; Table S6). Congenital heart disease (CHD) was present in 9 children and was more frequent in the CNV group than in the SNV group (7 vs. 2; *p* = 0.03); all defects were small without features of high pulmonary blood flow. One child underwent PDA device closure during neonatal ECMO for PPPHN. Sensorineural deafness, developmental delay, cognitive impairment and gastro‐esophageal reflux disease (GORD), occurred more frequently in the CNV group. Sensorineural deafness was not attributable solely to ECMO exposure as 4 of 5 affected children had no history of ECMO.

### 3.3 Histopathology

Lung tissue was available from 5 children (4 biopsies and one explant), sampled at a median age at sampling of 0.9 years (range 0.1 to 9.0; Table S7). All demonstrated abnormalities of lung growth, characterized by predominantly patchy alveolar simplification (Figure 3a,b). Bronchial abnormalities were present in 4 of 5 children and included bronchial epithelial and smooth muscle hyperplasia (4/5), with bronchiolisation of the airspaces around the bronchioles (1/5), and bronchial diverticula (1/5) (Figure 3d,e). Pulmonary interstitial glycogenosis was present in 3 of 5 children. Pulmonary arterial remodeling was variable, occurring in 3 of 5 children (Figure 3f–i), with plexiform lesions identified only in explanted lung tissue (Figure 3h,i). Pulmonary venous changes were observed in two children, including pulmonary vein engorgement and mild fibrous intimal thickening. Lipid‐laden macrophages and cholesterol clefts were identified in 4 of 5 children (Figure 3c), suggestive of possible aspiration. In the two children without pulmonary arterial changes, biopsy timing differed from the clinical course of PH: one biopsy, performed at 7 months, preceded the development od subsequent progressive PH, while the other performed at 9 years following haemodynamic improvement on triple therapy after diagnosis age 3.6 years.

**Figure 3:**
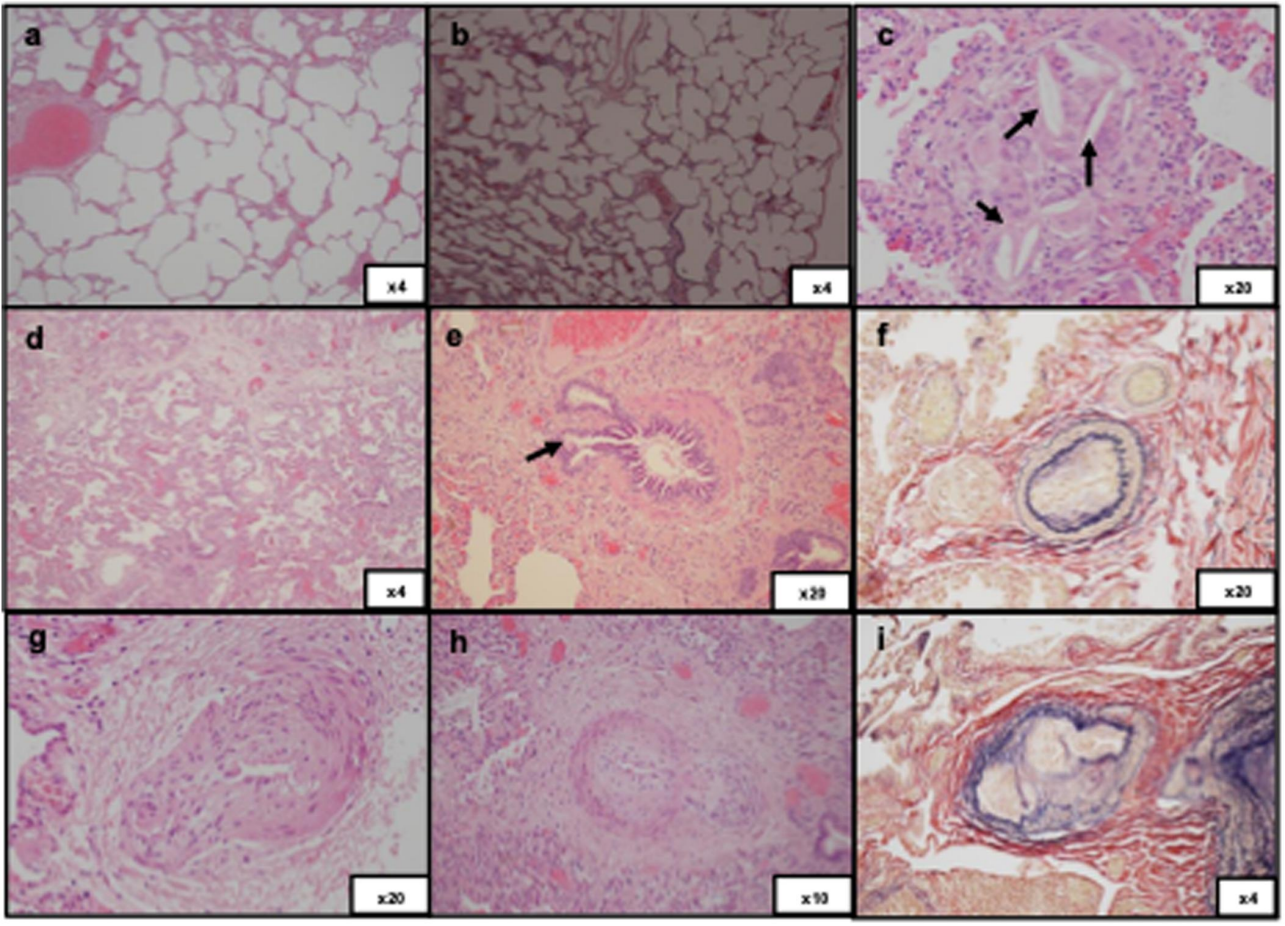
Representative histopathology specimens. Histology from lung explant in child age 1.6 years demonstrated diffuse alveolar underdevelopment characterized by enlarged simplified and rounded airspaces (*) with widened septa (a) and in a biopsy sample from a 7‐month‐old child with a patchy distribution of alveolar simplification (b). Cholesterol cleft deposits (c). Bronchial abnormalities including thickened, edematous bronchial walls with smooth muscle hyperplasia (d) and bronchial diverticular (arrow, e). Pulmonary artery remodeling included concentric intimal thickening with near occlusion of the vessel lumen (f) and fibrosis (g). Plexiform lesions (h, i) and pulmonary venous engorgement (a) was present in lung explant sample from child with end‐stage disease age 1.6 years. Haematoxylin and eosin stain (a–e, g, h) and Elastic Van Gieson stain (f, i). Magnification ×4 (a, b, d, i), ×10 (h), and ×20 (c, e, f, g). [Color figure can be viewed at wileyonlinelibrary.com]

### 3.4 Therapeutic Strategy and Response

Following initial assessment 9 (43%) children were managed with monotherapy, 7 (33%) with dual therapy and 4 (19%) with triple therapy, while PH resolved without long‐term pulmonary vasodilator therapy in one child (detailed below). Among treatment‐naïve children (*n* = 12), all demonstrated either improvement (50%) or stability (50%) in WHO‐FC following initiation of therapy, assessed after a median of 1.9 months (range 1.3 to 5.3).

By last follow‐up, treatment was escalated in nine children (53% of those not receiving maximal therapy) and de‐escalated in two children (10% of those receiving therapy). Three families declined intravenous prostacyclin therapy when this was offered. Nine (43%) children were treated with calcium‐channel blockers, which were always in combination with other pulmonary vasodilators. Nine children with ILD were treated with corticosteroids in addition to pulmonary vasodilators, as previously described by our group [27], notably with resolution of PH in one child following steroid therapy.

### 3.5 Long‐Term Outcomes

The median duration of follow‐up was 7.1 (range 0.7 to 17.2) years. Repeat haemodynamic assessment in 10 children demonstrated no significant change in mPAP or PVRi (see Table S4). Two children underwent Potts shunt creation, one child underwent bilateral lung transplantation at 1.6 years after initial assessment, and one child died at 6.7 years after first assessment. Transplant‐ or Potts shunt‐free survival was 100% (95% CI: 100% to 100%) at 1 year and 94.4% (95% CI: 84.4% to 100%) at 5 years. Outcomes were poorer in the CNV group compared with the SNV group (log‐rank *p* < 0.05, Figure 4b).

**Figure 4:**
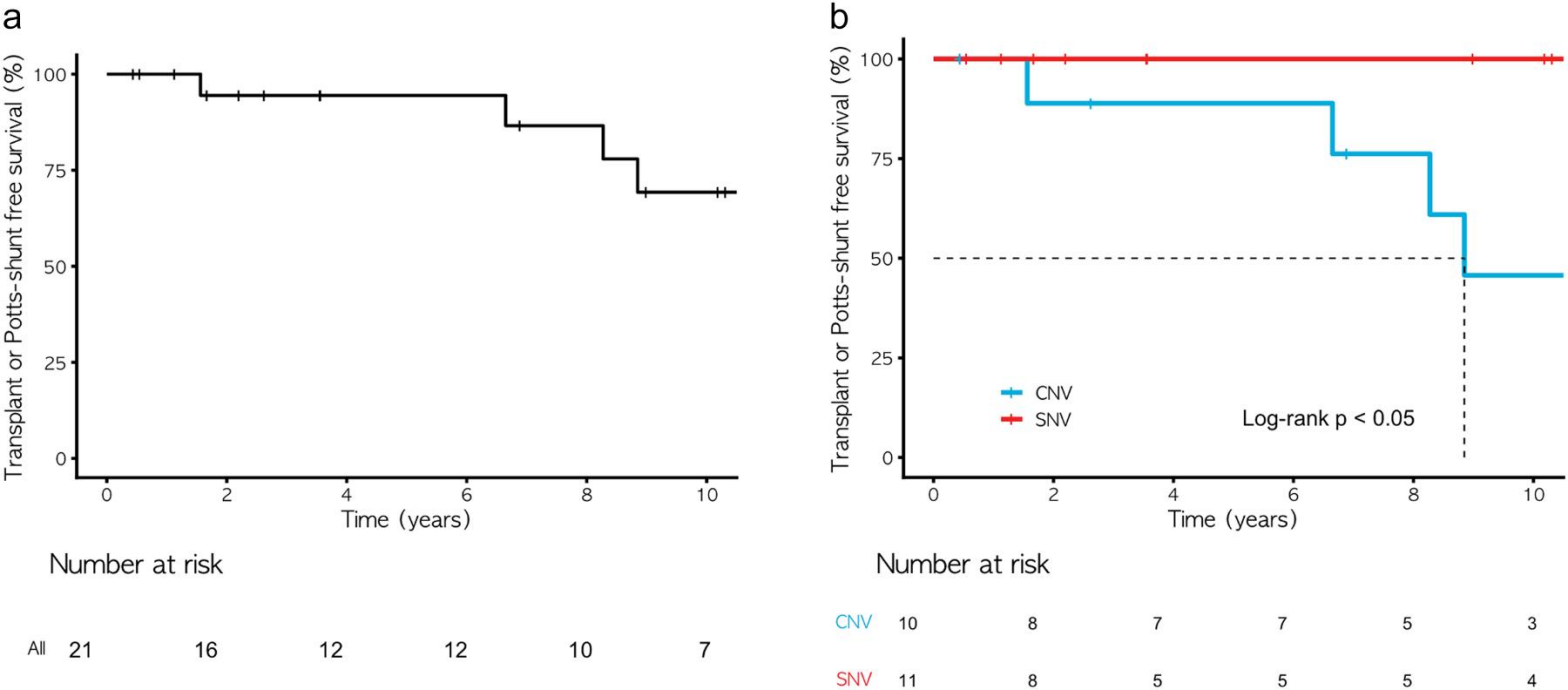
Kaplan‐Meier survival curves in (a) all children with *TBX4*‐associated pulmonary hypertension; (b) comparison of survival in children with a copy number variant (CNV) and children with variant confined to *TBX4* including single nucleotide variants, small insertions, deletions or duplications (SNV). Dashed line represents median survival time = 8.9 years. [Color figure can be viewed at wileyonlinelibrary.com]

## 4 Discussion

This study provides the largest and most comprehensive characterization of pediatric *TBX4*‐PH to date, defining it as a distinct syndrome combining pulmonary vascular disease with abnormal lung and lower‐limb development, often presenting in the neonatal or early infancy period with severe disease. Our cohort includes five previously unreported SNVs. Children carrying CNVs presented younger [13] and had more severe lung disease and multisystem disease than those carrying SNVs, who had higher rates of syncope and poorer somatic growth. Our findings extend prior observations and further support distinct phenotypic patterns associated with SNVs versus CNVs [12, 17, 29, 30]. These differences may partly reflect the involvement of additional developmental genes encompassed by CNVs, including the T‐box transcription factor *TBX2*, which may contribute to the cardiac, neurodevelopmental, auditory and other syndromic features observed and potentially influence clinical outcomes [12, 17, 29, 30].

Pulmonary vascular disease in *TBX4* syndrome is characterized by pre‐capillary PH with overlapping features of World Symposium on Pulmonary Hypertension Group 1 (pulmonary arterial hypertension) and Group 3 (PH due to chronic lung disease and/or hypoxia) [13, 14, 30, 31]. Baseline haemodynamics were comparable to previously reported pediatric *TBX4*‐PH cohorts [13, 32]. Despite the high prevalence of lung‐growth abnormalities, haemodynamic severity was comparable to Group 1 PH and exceeded that observed Group 3 PH [1]. Syncope was also more frequent (19%) than in children with Group 1 or Group 3 PH (11% and 1%, respectively) and in previously reported *TBX4* cohorts (11%) [1, 13]. In children, syncope may reflect labile pulmonary vascular resistance [33], and 42% of those tested demonstrated acute vasodilator responsiveness. Together, these findings suggest a phenotype of dynamic pulmonary vascular tone in *TBX4*‐PH, which may have implications for clinical monitoring and treatment.

Lung disease was common in our cohort (71%), with onset ranging from neonatal hypoxic respiratory failure and PPHN, to persistent hypoxemia beyond the neonatal period, and childhood‐onset ILD. *TBX4* is highly expressed during lung morphogenesis [11], providing a biological basis for early and often severe pulmonary involvement. Children with *TBX4*‐PH were diagnosed younger than those with Group 1 PH [1]. High rates of PPHN and neonatal ECMO requirement were consistent with previous reports [13], and were markedly higher than in our reported national pediatric PH cohort [1], further supporting a severe, early‐onset developmental phenotype. Three children were initially diagnosed with meconium aspiration syndrome, highlighting the importance of considering variants in *TBX4* in severe PPHN, even when another plausible cause is identified. Other features supporting underlying developmental lung disease included a high prevalence of neonatal pneumothorax, (42% vs. 21% in a previously reported *TBX4*‐PH cohort [13]), prolonged oxygen dependence, and radiological features including ground‐glass opacities and bronchial abnormalities. Lung disease was more severe in children carrying CNVs, consistent with previously reported patterns of lung disease in *TBX4* syndromes [34]. Our cohort did not include lethal lung developmental phenotypes previously reported in association with *TBX4* variants [15, 35, 36, 37, 38], likely reflecting survivorship bias; however, variable pulmonary expressivity is evident across pediatric and adult cohorts [13, 14, 30, 31, 32, 38]. Non‐coding regulatory variants affecting *TBX4* expression have also been associated with developmental lung phenotypes, further highlighting the genetic heterogeneity underlying the pulmonary phenotype [15, 39].

Most children in our cohort achieved clinical stability with pulmonary vasodilator therapy. In those with comorbid ILD, adjunctive corticosteroid therapy was associated with clinical improvement, as previously reported by our group [27]. Transplant‐free survival compared favorably with our historic idiopathic PH cohort (1‐ and 5‐ survival 89% and 75%, respectively [28]; Figure S1) and was [28]similar to a French cohort of both adult‐ and childhood onset *TBX4*‐PH (100% and 83%, respectively) [31]. These favorable long‐term outcomes suggest that clinical stability can be achieved with pulmonary vascular and lung‐directed therapies.

This study was limited by its retrospective design, spanning 24 years, during which clinical practice evolved, resulting in heterogeneity in management. Genetic testing strategies also changed over this period limiting our ability to accurately estimate disease prevalence.

*TBX4*‐PH challenges the traditional PH paradigm, reflecting dual contributions from pulmonary vascular and developmental lung pathology. We recommend genetic testing in children with idiopathic or familial pulmonary arterial hypertension, PH associated with CHD, or PH associated with unexplained developmental lung disease [40]. Given the multi‐system manifestations of *TBX4*‐syndrome, multidisciplinary assessment should be tailored to the individual phenotype, incorporating pulmonary hypertension, cardiology, pulmonology, clinical genetics, orthopedic, neurodevelopmental expertise as appropriate, to optimize care and outcomes. In children with 17q23 deletions encompassing *TBX4*, additional manifestations related to the wider multigene deletion should also be considered when tailoring clinical assessment and management.

## 5 Conclusion

Our findings demonstrate that variant class is associated with distinct clinical phenotypes and disease trajectories in pediatric *TBX4*‐PH. Children with CNVs present earlier, have more severe lung disease and multisystem involvement, and experience poorer outcomes compared with those carrying SNVs. Consideration of variant class may therefore aid prognostication and inform surveillance and management strategies in pediatric *TBX4*‐PH.

## Author Contributions

**Cara Morgan:** conceptualization, investigation, writing – original draft, methodology, writing – review and editing, formal analysis, data curation. **Alistair Calder:** writing – review and editing, data curation, formal analysis. **Matina Prapa:** formal analysis, writing – review and editing. **Thivya Sekar:** writing – review and editing, formal analysis. **Andrew Constantine:** writing – review and editing, data curation. **Tomas Woodgate:** data curation. **Annabelle Barnes:** data curation. **Eimear McGovern:** data curation. **Paul Aurora:** writing – review and editing. **Rossa Brugha:** writing – review and editing. **Sadia Quyam:** writing – review and editing. **Andrew Bush:** writing – review and editing. **Shahin Moledina:** conceptualization, supervision, writing – original draft, writing – review and editing.

## Ethics Statement

This study was approved by the NHS Health Research Authority and the associated Research Ethics Committee (REC reference: 17/LO/0008) under the approval titled “Use of routine Great Ormond Street Hospital data for research.” The study used anonymized clinical data, allowing individual consent to be waived, and was conducted in accordance with the principles of the Declaration of Helsinki and applicable UK regulatory requirements.

## Funding

The authors have nothing to report.

## Conflicts of Interest

Cara Morgan, Alistair Calder, Martina Prapa, Thivya Sekar, Andrew Constantine, Tomas Woodgate, Anabelle Barnes, Eimear McGovern, Paul Aurora, Rossa Brugha, Sadia Quyam, Andrew Bush, and Shahin Moledina have nothing to declare.

## Take Home Message

Children with *TBX4* copy number variants have earlier presentation, more severe lung and multisystem disease, and worse outcomes than those with single nucleotide variants, supporting genotype‐informed risk stratification and tailored management.

## Supporting information


Supporting File

Supporting File

## Data Availability

The data that support the findings of this study are available from the corresponding author upon reasonable request.
