# Supplementary material for: Variant Class–Specific Phenotypes and Outcomes in Pediatric TBX4‐Associated Pulmonary Hypertension
Source: Pediatr Pulmonol. 2026 Oct 6;61(10):e71857. doi: 10.1002/ppul.71857 (PMC13640964; doi:10.1002/ppul.71857)
Supplement: Supplementary file 1 [file PPUL-61-0-s001.docx]

**SUPPLEMENTARY DATA**

**Figure S1:** Kaplan-Meier survival curves for transplant-free survival. a) all children with TBX-4 PH: transplant-free survival was 100% (95%CI: 100% to 100%) at 1 year and 94.4 % (95%CI: 84.4 % to 100%) at 5 years. b) Comparison of survival in children with a copy number variant (CNV) and variant confined to TBX4 including single nucleotide variant*s,* small insertions, deletions or duplications (SNV).

**a)**

**b)**
